# Reporting Chemical Data in the Environmental Sciences

**DOI:** 10.1021/acsenvironau.5c00034

**Published:** 2025-07-29

**Authors:** Sivani Baskaran, Parviel Chirsir, Shira Joudan, Raoul Wolf, Evan E. Bolton, Paul A. Thiessen, Emma L. Schymanski

**Affiliations:** † 72989 Norwegian Geotechnical Institute (NGI), 0484 Oslo, Norway; ‡ Luxembourg Centre for Systems Biomedicine (LCSB), University of Luxembourg, 6 Avenue du Swing, 4367 Belvaux, Luxembourg; § Department of Chemistry, 3158 University of Alberta, Edmonton, Alberta T6G 2G2, Canada; ∥ National Center for Biotechnology Information (NCBI), National Library of Medicine (NLM), National Institutes of Health (NIH), Bethesda, Maryland 20894, United States

**Keywords:** chemical information, data, FAIR, open access, accessibility, reproducibility, environmental science

## Abstract

Environmental sciences, including environmental chemistry and toxicology, are highly interdisciplinary fields that integrate researchers with various backgrounds and expertise. This interdisciplinary aspect is critical to addressing issues of chemical pollution, environmental sustainability, and health. However, a standardized method for reporting chemical data is needed to address these issues effectively. This becomes increasingly important as both the number of chemical structures and our reliance on and use of computational analysis and cheminformatics tools grow. This paper provides background, examples, and recommendations on how to report chemical data in a findable, accessible, interoperable, and reproducible (FAIR) manner within environmental science disciplines. Ultimately, the goal is to broaden the scope and applicability of environmental research to help the entire community tackle the issues of chemical pollution and sustainability in a comprehensive manner.

## Graphical abstract

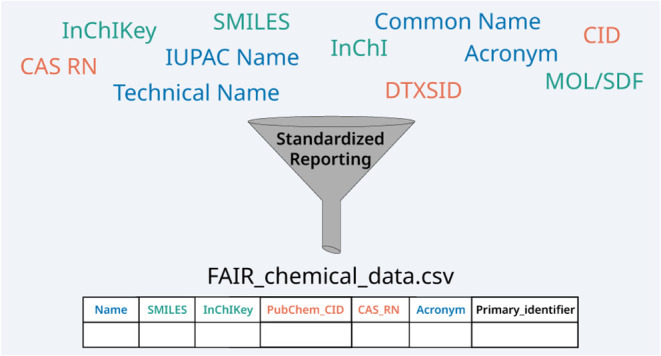

## 1 Introduction

Environmental science is an interdisciplinary and collaborative field that brings together expertise and knowledge from a wide range of disciplines, including analytical, environmental, organic, and physical chemistry, toxicology, and biology, plus atmospheric, health, social, and data sciences. One key aim of this scientific intersection is to understand the behavior of chemicals in the environment and their interactions with humans and other organisms. However, this objective is sometimes impeded by data accessibility issues. Although the largest chemical databases contain hundreds of millions of chemicals, they are known to be incomplete, as many analytical signals detected in the environment cannot be matched to any chemicals in these databases.^1–3^ While notable progress has been made to improve the accessibility of scientific knowledge via the rise of open access publishing options, less attention has been given to making the chemical data associated with environmental studies findable, accessible, interoperable, and reusable (FAIR).^4^ Building on recent recommendations on FAIR chemical data,^5^ this perspective aims to provide background, examples, and finally, guiding principles for reporting chemical data in environmental sciences.

A chemical’s identity is crucial information in the chemical and environmental sciences. This information is used to distinguish chemicals listed in global chemical registries, determine their properties, apply for patents, describe use applications, and so on. In 2003, Glüge et al.^6^ reviewed 8590 substances from the European Chemicals Agency (ECHA) database and cross-referenced them with multiple chemical databases. They identified several issues in the reported data due to inconsistent identifiers and missing stereochemical information.^6^ An earlier 2008 ^7^ analysis of multiple databases found errors in the structure of chemicals reported relative to other chemical identifiers.^7^ As informatics approaches become more accessible, errors such as these will be increasingly exacerbated due to “cross-pollination” between different databases as data are copied and shared across platforms. However, errors are not necessarily always errors per se, but can arise due to discrepancies in how the data was collected and processed. Whether the data are obtained from the field, lab, or through computational methodologies, the data presented in scientific publications holds immense value to other researchers. Publications are most useful to the chemical community when the chemicals described are well-defined using standard and precise identifiers.

For the purposes of this article, it is important to first define the concepts “chemical compound” and “chemical substance”. This is illustrated with examples in Figure 1. The International Union of Pure and Applied Chemistry (IUPAC) definition of a molecule is a neutral entity, made up of at least two atoms.^8^ In this article, a chemical compound is composed of a single entity, made up of at least two atoms, which collectively has either a neutral or charged state, and has specific physical–chemical properties (see Figure 1). IUPAC’s definition of a chemical substance is a unit of matter with a constant composition that can be characterized by the molecules, formula units, and atoms it is made up of.^9^ By these definitions distinct structural and stereoisomers are considered different chemical compounds (see Figure 1, compounds A–E); while a nonstereochemistry specific entity with stereocenters (see Figure 1, substance A, also Figure SI 1), mixtures, Unknown or Variable composition, Complex reaction products and/or Biological materials (UVCBs), or polymers are considered to be substances. Thus, with these definitions, (1) both substances and compounds have distinct physical–chemical properties, (2) chemical compounds can also be chemical substances, but (3) not all substances are chemical compounds. Importantly, the definitions of chemical compounds and substances used by published databases and systems can differ from the definitions presented here and from each other.

**Figure 1:**
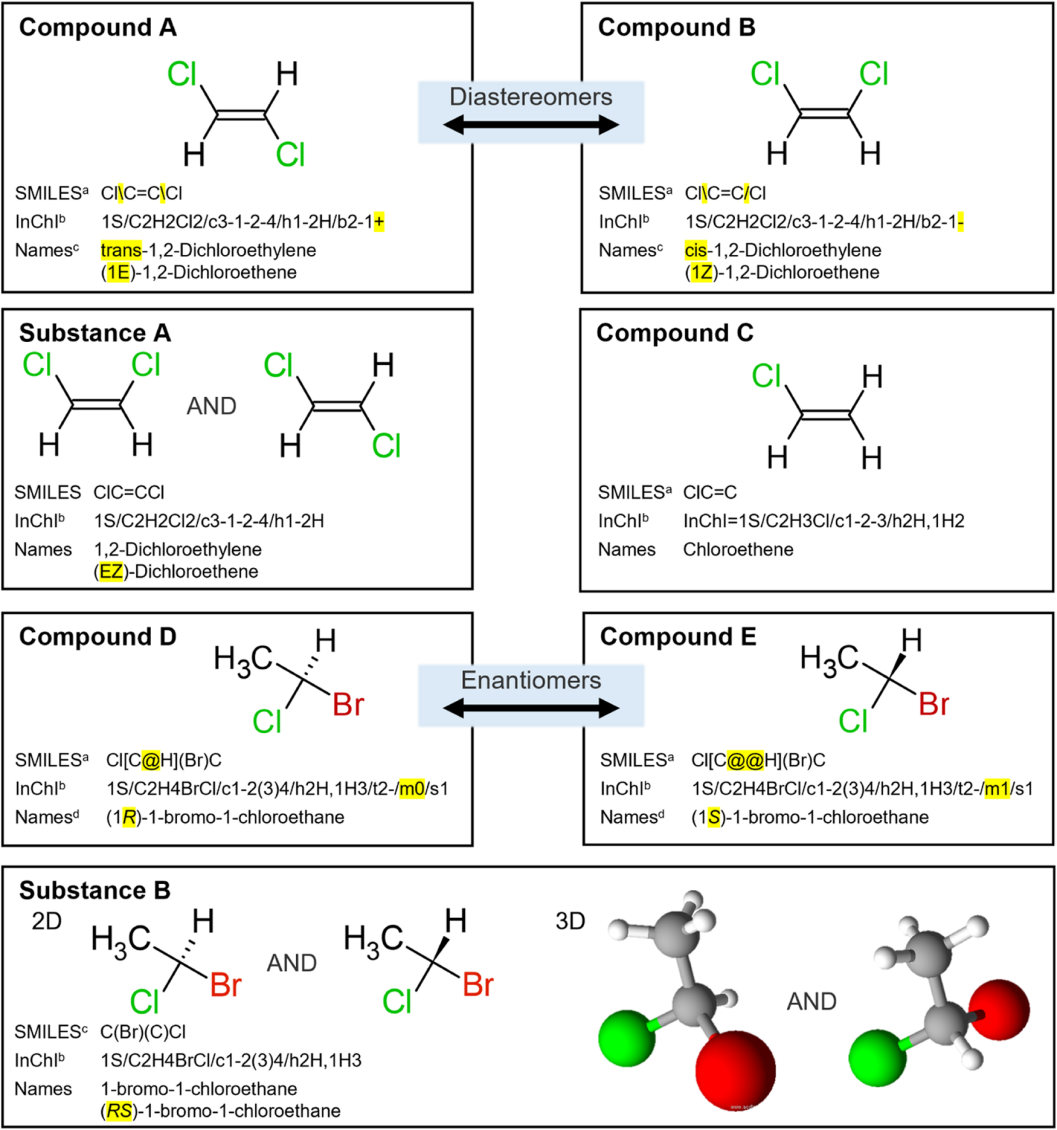
Examples distinguishing the differences between compounds and substances as defined in this work. SMILES, InChIs, and some names are provided for each chemical. Distinct differences in these identifiers between similar substances are highlighted in yellow. Compounds A and B are diastereomers, stereoisomers that are not mirror images of each other. Compounds D and E are enantiomers, stereoisomers that are mirror images of each other. Substance B can also be depicted as a single 2D structure using a single bond instead of hashed or wedged bonds. Identifiers were generated manually or taken from ^a^ ACD/Labs ChemSketch,^10^ ^b^ Open Babel,^11^ ^c^ CAS Common Chemistry,^12–14^ ^d^ PubChem.^15,16^ Structures were generated using ACD/Labs ChemSketch.^10^

This article begins by reviewing commonly used chemical identifiers in environmental sciences: chemical names, database identifiers such as CAS Registry Numbers (CAS RN), and single-line text-based structural chemical representations such as Simplified Molecular Input Line Entry System (SMILES). The pros and cons of the various identifiers are discussed, along with how and where to obtain this information and the role that names, structures, and numerical identifiers play in the reporting of data. Finally, recommendations are provided on what information should be given when reporting chemical data and associated results in the environmental sciences, based on the FAIR chemical guidance.^5^

## 2 Chemical Data

Regardless of the type of research undertaken, the reported data are often underpinned by the chemical information on the substances studied, i.e., information used to identify the chemical. Typical chemical identifiers reported include a common name (e.g., DDT) or CAS RNs (e.g., 50-29-3), but these reporting conventions can be prone to errors and challenges. Table 1 includes an overview of various ways to exchange chemical information.

**Table 1:** Description of Some Common Chemical Identifiers

chemical identifier	description
CAS RN	**CAS** **R**egistry Number assigned by the American Chemical Society; CAS RNs have a standardized three-block format containing 2 to 7 digits, 2 digits, and 1 digit, respectively; the final digit is used as a check digit that can be used to verify valid CAS RNs^17^
CID	**C**ompound **ID**entifier used in PubChem
common name	colloquially used term/acronym to refer to the chemical; often does not describe the structure
DTSXID	**D**SS**T**ox **S**ubstance **ID**entifier used in the Environmental Protection Agency’s CompTox Dashboard
InChI	**In**ternational **Ch**emical **I**dentifier—a standardized text-based identifier for chemical structures that encodes molecular information
InChIKey	a three-block hash key of the InChI (skeleton-stereochemistry-charge) with 27 characters (14-10-1); the second block also includes isotopic information, the version used, and whether the original InChI is standardized^18^
IUPAC Name	a name derived using **I**nternational **U**nion of **P**ure and **A**pplied **C**hemistry (IUPAC) naming conventions
MOL/SD file	an MDL Molfile (*MOL*) contains information about the atoms, bonds, connectivity and coordinates of a single molecule. Multiple entries can be stored in a structure data (SD) file (*SDF*), separated by “$$$$”
SMILES	**S**implified **M**olecular **I**nput **L**ine **E**ntry System—a line notation for describing the structure of chemical species using short ASCII strings
synonyms	other names, variations, spellings, simplifications, or abbreviations of the other names
technical name	name used and registered by companies in patents or products

### 2.1 Chemical Names

Variations in naming conventions can create challenges in matching and correctly identifying chemicals. Starting with DDT, domain-specific knowledge would lead to the assumption that the acronym is referring to *p*,*p*′-DDT or 4,4′-DDT, but without knowledge of common or discipline-specific naming practices, it could also be misinterpreted as *o*,*p*′-DDT or 2,4′-DDT (Table SI 1, rows 8 and 9). Next, polychlorinated biphenyls (PCBs) are a well-established group of chemicals, with many different naming systems used to describe single congeners. A congener is used to describe chemicals with similar structure and/or properties. For PCBs, there are 209 unique congeners.^19^ Box 1 includes some examples of names used for one hexachlorobiphenyl congener, PCB 150, revealing issues of order, separators (“-” vs “ ”), and various naming conventions. Although none of these names are incorrect, an exact text match will work only if the names in the database and search query match perfectly.

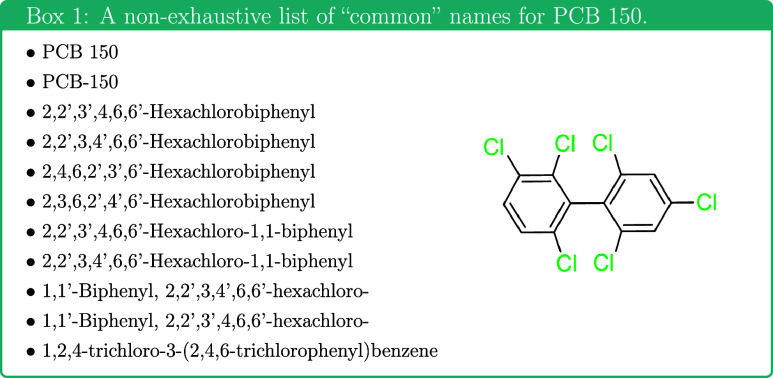



The stereochemistry specification is extremely important in some environmental chemistry and toxicology applications, where 3D conformations of chemicals are relevant. Different stereoisomers can have different physical–chemical properties, and thus, their environmental and biological implications can differ. A study testing the toxicity of 6PPD-quinone found that the *S* enantiomer was more toxic than the racemic mixture or the *R* enantiomer.^20^ Hence, the naming and identification of specific isomers have chemical, environmental, and regulatory importance. More details regarding differences in isomerization in chemical structures are included in Section SI 2 and Brunning.^21^

Another example is hexabromocyclododecane, also commonly referred to as HBCD or HBCDD. The name hexabromocyclododecane indicates a cyclic chain of 12 carbons with 6 bromine substitutions. Under the Stockholm Convention, HBCD is listed as a persistent organic pollutant and supporting documents primarily refer to 6 of the most common isomers of 1,2,5,6,9,10-hexabromocyclododecane,^22,23^ which has 16 different stereoisomers (see Section SI 2). When multiple stereoisomers are possible, each isomer is assigned a Greek letter; enantiomer pairs are assigned the same letter and sometimes differentiated using + or – based on their optical activity.^24^ While useful as a quick reference, these letters pose many challenges and can produce errors when working with computational software that may not recognize these characters. It is also important to note that the locations of the bromine substitutions are not specified in the original common name “hexabromocyclododecane”; thus, outside the regulatory context of the Stockholm Convention, HBCD can represent 77 possible structural isomers, assuming 6 single bromine substitutions. This count does not include possible stereoisomers for each of the structural isomers.

In some cases, there are preferred English spellings for chemicals depending on the region, for example, endosul*ph*an^25^ vs endosul*f*an.^26^ The use of *f* over *ph* is now recommended by IUPAC as the proper name for sulfur compounds.^27^ The distinction between phosphorus (noun) and phosphorous (adjective) in English has also led to much confusion and has been used interchangeably in chemical naming systems.^28–30^ However, not all publications and documents are in English and additional identifiers are needed to complement a chemical name. The problems with using names to identify chemicals are exacerbated for large, novel chemicals, making matching and identifying papers referencing the same chemical increasingly difficult.

In chemistry, there are standardized naming processes defined by IUPAC, which are described extensively in the Blue Book^31,32^ for organic chemistry, the Red Book for inorganic chemistry,^33^ and the Purple Book^34^ for polymers. Using these guidelines, only one IUPAC name should exist per substance. IUPAC has also consolidated shorter guidelines that are available online;^35–40^ however, the use of such guidelines can be cumbersome and time-consuming. There are tools for generating the IUPAC names using the structure of a compound (e.g., ChemDoodle^41^) and parsers that can provide a structure of a chemical given the IUPAC name (e.g., Open Parser for Systematic IUPAC nomenclature^42,43^ ). Despite IUPAC’s best intentions, there are issues in reading and writing IUPAC names. Taking *p*,*p*′-DDT again, the IUPAC name for this molecule is reported to be 1,2,4-trichloro-3-(2,4,6-trichlorophenyl)­benzene by PubChem^44^ and CACTUS^45^ and 1,1′-(2,2,2-trichloroethane-1,1-diyl)­bis­(4-chlorobenzene) by CompTox^46^ and ChemSpider.^47^ Complicated structures have further issues, such as the superscripts in the IUPAC name 1,3,5,7-tetrazatricyclo­[3.3.1.1^3,7^]­decane for methenamine.^48–50^ While these standardized names can help determine the exact molecular structure of a chemical, they are not easy to generate or interpret for large molecules and, as demonstrated, are not completely interoperable.

### 2.2 Database Identifiers

Different databases and registries have introduced many numerical identification systems to index their entries and reduce these types of errors, including, CAS RNs (American Chemical Society),^17,51^ Compound and Substance IDentifiers (CIDs and SIDs respectively, PubChem, United States National Institute of Health),^52,53^ DTXSIDs and DTXCIDs (CompTox Chemistry Dashboard, United States Environmental Protection Agency),^54^ ChEMBL IDs (ChEMBL Database, European Molecular Biology Laboratory),^55^ ChEBI IDs (Chemical Entities of Biological Interest Database, European Molecular Biology Laboratory),^56^ and ChemSpider IDs (CSID, Royal Society of Chemistry).^57^ Because these identifiers are specific to a database, they typically map to a single entry within the database; however, only entries that are already within these respective resources are assigned an identifier. In many cases, the databases cross-reference each other, and almost all attempt to include alternative identifiers in their list of chemical identifiers. Some identifiers, such as CAS RNs, are not readily available unless they have been previously published or made available via CAS Common Chemistry^58^ and can only be authoritatively identified using proprietary tools such as SciFinder.^59^ Additionally, database identifiers may be deleted, replaced, or deprecated over time. As a result, a chemical structure may have two or more identifiers. For example, *p*,*p*′-DDT has a current (50-29-3) and deleted (1081843-15-3) CAS RN that can be found in different databases and publications. While one database may consider one name or structure to be unique, another may split the substance into multiple entries to account for stereochemistry, isomerization, or tautomerization. This can often depend on the curation and standardization algorithms used in the databases. This is discussed further in Section 2.4.

In other instances, the name or chemical referenced in a publication is not included in any chemical databases, or there may not be a numerical identifier assigned to a particular structure or available on an open-access platform. Property data for 6-OH BDE-68, a polybrominated diphenyl ether congener (PBDE 68) with an additional hydroxy group, was published by Liu et al.;^60^ while this chemical (at the time of writing) appears only on PubChem (CID: 101542767) the name used by Liu et al. is not used or included under PubChem’s synonyms list. Shorthand names such as 6-OH BDE-68 must be identified and manually added to any database and registries, which can take time or may never occur unless specifically requested.

It is not uncommon for environmental scientists to work with ionizable substances. One excellent example is perfluorooctanesulfonate, which is a charged ionic form of perfluorooctanesulfonic acid (PFOS). With a charge of −1, perfluorooctanesulfonate exists as a salt with lithium and potassium as common counterions (Figure SI 5). Each salt is often assigned a unique identifier within databases and has distinct structural identifiers; however, these entries do not account for all possible counterions. If the salt is purchased directly from a supplier, it can be useful to specify which salt is being used and specifically identify the analyte or ion of interest. The importance of the salt is relative to the methodology; for example, in toxicology, it is important to use the correct molecular weight when weighing a salt and converting it to concentration units of PFOS in a toxicology study, whereas in environmental water samples, PFOS exists in a dissociated state.

When working with spreadsheets, CAS RNs pose a particular problem: they can be misinterpreted as a date by Microsoft Excel. A good example of this is dichloromethane; its CAS RN 75-09-2 can, for example, be automatically converted to September 2, 1975. This error occurs whenever the first set of digits in the CAS RN contains 2 digits or contains 4 digits which can be read as a year (e.g., 75 or 1975) and the second set of digits contains two digits between 01 and 12. The genomics field experiences a similar problem with some gene symbols (e.g., MARCH1); this has since been addressed by updated nomenclature guidelines and renaming symbols that were affected during data handling.^61^ Analyses of genomic papers indicate that approximately 20% of gene names were erroneous in papers that contained supplementary Excel files^62^ and approximately 30% of all publications in the field contained these types of errors.^63^ No comparable study examining CAS RN errors within environmental science or chemistry publications was found. In 2023, Microsoft Excel introduced an option to remove the automatic date format conversion process; however, preventing these errors relies on researchers working with this type of data to toggle on this functionality before using or opening files that contain CAS RNs and it is not guaranteed to work.^64^ When adding CAS RNs to a Microsoft Excel document, it is possible to avoid automatic conversion of the CAS RN to a date by introducing an apostrophe before the CAS RN (e.g., '75-09-2). However, this applies only to *XLS*/*XLSX* files and such additions will be lost if the file is saved as a *CSV* and reopened as an Excel document and conversely may be retained as part of the CAS RN when opened by other programs or software.

### 2.3 Structural Representations

There are multiple methods for describing chemical structures that provide varying levels of detail. Single-line text-based notations that are used to describe chemical compounds include SMILES, InChI, and InChIKeys.

SMILES is the most human-readable format; with a bit of practice, it is possible to generate and read the SMILES of simpler and smaller compounds without the use of computational tools.^65^ SMILES can come in a variety of flavors, including standardized, canonical, QSAR-ready, MS-ready, universal, kekulized, etc. The terms are not always used consistently between toolkits and databases, and in the case of MS- and QSAR-ready SMILES, there can be different or reduced levels of specificity depending on the purpose and use case.^66,67^ By definition, SMILES can be created from multiple starting points, so the order of the atoms may change, and a single structure can have many equivalent and valid SMILES. In some forms, the specificity of the stereochemistry (e.g., relative positions or aromaticity) or dative bond pairs are excluded from the structure, an issue sometimes undetected by those unfamiliar with SMILES, which has led to much confusion in reporting structures (see Section SI 3 for additional information and examples).

Returning to the 1,2,5,6,9,10-HBCDD example from Section 2.1, the nonstereospecific SMILES should be associated only with 1,2,5,6,9,10-HBCDD without defined stereochemistry, but SMILES notation can be used to differentiate between all 16 isomers (see Table SI 1 and Figures SI 1 and SI 2). As a rule of thumb, it is good to check if the SMILES produces the same structure with the same level of specificity one would expect by using free chemical drawing software such as ACD/Labs ChemSketch,^10^ CDK Depict,^68^ Smi2Depict,^69^ or MolView.^70^ Other widespread tools such as Marvin/MarvinSketch^71,72^ and ChemDraw^73^ can also be used for the same purpose. SMILES is one of the most common input formats for QSAR models in environmental chemistry and toxicology (in part due to its ability to be included within a spreadsheet); however, there are some issues to keep in mind. For some models, different SMILES for the same chemical can produce different results (e.g., EPISuite^74^), while others perform an internal standardization process (e.g., OPERA^75^). Thus, it is better to have prepared all SMILES data in the same way before using them as input for any QSAR or predictive tool to ensure consistent results.

The International Chemical Identifier or InChI was developed by IUPAC.^18,76^ Unlike SMILES, InChI is calculated using a standardized algorithm, and thus one InChI exists per structure. This enables InChIs to be used as identifiers. However, InChIs are generally much longer than SMILES and contain several special characters (commas and semicolons) that can cause parsing issues. For large molecules, the length of InChIs can also lead to truncation errors if they exceed database limits.^18,77^ To alleviate these issues, the hashed form InChIKey was introduced to serve as a machine-readable identifier. An InChIKey can be generated from an InChI, but an InChI can not be computed from an InChIKey.^18,76^ If an InChIKey is the only information available, a database or look-up table containing the InChIKey is required to retrieve the chemical’s structure. As such, providing an InChIKey without its corresponding InChI or SMILES can be problematic, especially if one describes a novel structure.

InChIs have different layers containing structural information. The standard form, denoted with “1S/”, is calculated with standard settings and thus always comparable (see Heller et al.^18^ for more details). The use of a nonstandard InChI, denoted with “1/”, indicates that nonstandard (and therefore not always comparable) settings were used and are sometimes needed to capture advanced stereochemistry or tautomers. Each layer provides different information about the chemical structure, such as atom connectivity, stereochemistry, and charges. The standardized InChI produces a unique standard InChIKey, denoted by the “SA” at the end of the second block. Nonstandard InChIKeys are denoted by “NA” at the end of the second block. The standard InChI does not always differentiate different tautomers; in these cases, nonstandard InChIs can be used to define specific tautomerization. Nonstandard InChIs may also be necessary to define nonstereospecific structures. In the case of a structure with relative stereochemistry, the SMILES and standard InChI may reflect the structure of one of the enantiomers; the use of a nonstandard InChI and InChIKey allows for the specificity of relative-stereoisomerization (Table SI 1, see Yerin^78^ for additional examples). Since standard InChIs are produced using fixed rules, SMILES generated from standardized InChIs may not always provide the most environmentally relevant structure. For example, atrazine has five tautomers (Table SI 2), which are all described by the same standardized InChI string. Thus, if only one single-line notation is used, SMILES is often preferred to define the structure with the necessary stereochemistry, while InChI and InChIKey can be used to verify and identify the SMILES.

The structure of a chemical can also be expressed visually with lines and atom symbols. Drawing the structure can help reduce the ambiguity of other chemical identifiers, and there are various ways a structure can be drawn and annotated (see Figure SI 3). For simple structures, general drawing tools can be used to create and share the structure as an image file. However, using chemistry-specific tools for drawing substances is generally much easier, reducing the potential for errors, increasing interoperability—as the shared file can be easily modified and saved in recognized exchange formats—and they are generally better equipped to handle large and more complex substances. These structures can be exported as an image file or shared using specific file types, such as *SK2*, *SKC*, *CDX*, or *CML*, which can only be read by specific tools, as discussed later. *MOL* (MOLfile) contains a connection table with information about the bonds and can include 2D or 3D coordinates of a chemical and its physical–chemical property data.^79^ An Structure Data File (*SDF*) can contain multiple *MOL* files^79^ and thus can contain 2D or 3D chemical data for any number of chemicals. Both *MOL* and *SDF* files are machine-readable and, to a certain extent, human readable. There are two versions of *MOL* and *SDF* files, which are used interchangeably but look notably different.^80^ The original format was developed by Dalby et al.^79^ and current standards for v2000 and v3000 are set by BIOVIA.^81^ v2000 is more widely used, however v3000 can handle more complex chemistry.^80,81^

While there are many challenges with using structural representations, their use in combination with classical chemical identifiers, including names and database identifiers, can help reduce errors in chemical identification and identify erroneous entries. As previously noted, databases can have erroneous data where names and structures do not match.^6,7^ These errors are often due to a lack of available information regarding the exact structure of a compound, and if sufficient chemical structure information is provided with chemical names (and vice versa), errors can often be detected and corrected.

### 2.4 Structural Complexities

The structural representations discussed above work well to describe many compounds and substances. However, chemical information on ambiguous substances, mixtures, UVCB substances, and polymers is more challenging to manage in databases and for use with cheminformatics tools.

A substance that is ambiguously described may be lacking information regarding specificity of bond locations, e.g., trichloropyrene (CAS RN: 83690-29-3) or HBCD (CAS RN: 25637-99-4 ^82^ and DTXSID8025383 ^83^) or have ambiguous stereochemistry (e.g., 6PPD-quinone or 1,2,5,6,9,10-HBCDD; see also Figure 1, substances A and B). Gobbi and Lee^84^ note that when the stereochemistry of a substance is ambiguous, the substance should be considered to be a mixture of the stereoisomers. These ambiguous substances are often treated as singular compounds, and the need for specificity in the stereochemistry is not always apparent; it is therefore beneficial to be as specific as possible when reporting data. Stereoisomers can sometimes be identified by searching databases and registries for the substructure or similar substances (see Section SI 2); alternatively, some chemistry drawing tools such as ACD/Labs ChemSketch have methods for generating all possible stereoisomers for a given structure.^10^ In the case of mass spectrometry, stereochemistry information is rarely available, and if there is no way of determining it otherwise, reporting structures without stereochemistry is sometimes the best approach to match the given information.

Some mixtures, such as racemic mixtures, are composed of equal quantities of two enantiomers (see Figure 1, substance B). In other instances, the ratio of constituents or the composition of the mixture is unknown and the substance could be classified as a UVCB. Take for example the UVCB, “Technical chlordane”, which is a commercial mixture that includes chlordane isomers and related compounds including isomers of nonachlor, *cis*-, and *trans*-nonachlor.^85^ Previous analysis identified 120 components of the technical mixture, which includes 13 nonachlor C_10_Cl_9_ isomers.^86^ The most common isomers, *cis*-nonachlor and *trans*-nonachlor, are found in some databases, individually or combined with a generic structure (Table SI 1). It is difficult to combine the details for all of these “technical chlordane” substituents into one identifier, and in many cases, not all substituents of the UVCB are known. Lai et al. provide specific recommendations for reporting and describing UVCBs.^87^ Notably, they suggest providing as much detail as possible regarding the substance and suggest the use of extended SMILES, i.e., SMILES with additional layers of notation capturing structural unspecificity, and future use of Mixture InChIs (MInChIs), which is an IUPAC project in development.^87,88^ While no system for describing UVCBs is touted to be perfect, providing as much information as possible increases the interoperability of the chemical data and could allow future researchers and developments to retroactively improve the identification of these UVCBs in the future.

Polymers can also be difficult to describe or model.^89,90^ Audus and De Pablo note that polymers are often not made up of single entities but can be composed of different branches and contain chiral or multiple monomers.^90^ Some databases also assign identifiers (e.g., CAS RN), to specific monomers, however, this does not take into account the complexity of synthetic polymers which can be composed of various combinations of unique monomers.^90^ Japan’s National Institute for Materials Science has published a database of polymers (PoLyInfo) with its own numbering system and more recently development of an ontology system for polymers.^91,92^

## 3 Finding and Generating Chemical Data

It is difficult to obtain chemical identifiers without prior knowledge of the available chemical resources. Information can be obtained from a wide range of databases, journal articles, reference books, encyclopedias, safety data sheets, chemical registries, and chemical data tools. Depending on the preference and research scope of the user, some sources may be considered more reliable than others, yet no data source is comprehensive or complete. Access to data sources may be limited due to commercial licensing or intellectual property rights, while the accuracy of the data is dependent on the level of curation and reliability of the reported data or tool. These resources contribute immense value to the environmental sciences. Table 2 provides a nonexhaustive list of open resources available for obtaining chemical identifiers; while some of these resources provide additional information about the chemical structures, this table focuses on the availability of chemical identifiers (names, database identifiers, and structural representations). The availability of information depends on the compound or substance and the database. There may be instances where the reported CAS RN is a deprecated or deleted number, or different synonyms and spellings are used in different data sets. More resources can be found on the Wikipedia Page for Chemical Databases.^93^

**Table 2:**
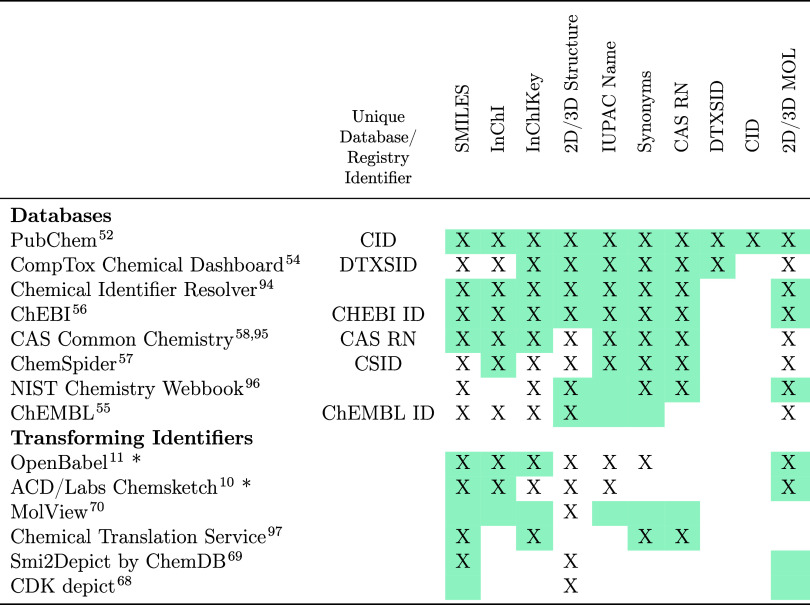
Selection of Free Tools Available for Finding or Calculating Different Chemical Identifiers^a^ ^,^ ^94–97^

a “X” indicates that the tool can provide or produce these data; shaded cells indicate what input values or formats are accepted by the tool. * indicates that the tool is a downloadable program.

## 4 Current Reporting Guidelines

Current author guidelines for reporting chemical data in publications are inconsistent and can differ even between journals from the same publisher. Publishers play a key role in facilitating data sharing and encouraging the application of FAIR principles.^4^

As of early 2025, for the American Chemical Society (ACS) series of journals relating to environmental sciences, the journal guidelines recommended the use of SI units and the inclusion of IUPAC or International Union of Biochemistry and Molecular Biology (IUBMB) substance names. This includes Environmental Science & Technology (ES&T),^98^ Environmental Science & Technology Letters,^99^ ACS ES&T Engineering,^100^ ACS ES&T Air,^101^ ACS ES&T Water,^102^ ACS Environmental Au,^103^ and Environment & Health.^104^ ES&T,^98^ ES&T Letters^99^ and ES&T Air^101^ further recommend including chemical names or composition in the first mention. Environment & Health^104^ and ACS Environmental Au^103^ both recommend including trade names at first mention. These guidelines are in contrast to those of ACS’s Journal of Chemical & Engineering Data (J. Chem. Eng. Data) guidelines,^105,106^ which require authors to provide the IUPAC name, CAS RN and 2D structural identifiers (e.g., SMILES, InChIs) for all chemicals studied. For experimental data, J. Chem. Eng. Data further require the source of the chemicals and details regarding the purity of the substance.

The Society of Toxicology and Chemistry (SETAC) publishes two journals, Environmental Toxicology and Chemistry and Integrated Environmental Assessment and Management. Neither journal has any requirements or suggestions for reporting chemical data and identifiers in their author guidelines as of 2024.^107,108^ However, SETAC has published a Technical Issue Paper on “Recommending Minimum Reporting Information for Environmental Toxicological Studies”.^109^ While the focus is not only on chemical data, the authors make a clear call for increasing transparency and improving reporting practices.^109^ Ågerstrand et al. further suggest that the technical name, CAS RN, purity, source, and physical–chemical properties of the chemicals used in the test be reported.^109^ SETAC also has a Data Transparency Policy, which requires authors to include a data availability statement and encourages authors to provide data on open repositories.^110^

Elsevier provides no suggestions for reporting chemical information in their author guidelines for Chemosphere,^111^ Environment International,^112^ Environmental Pollution,^113^ Environmental Technology & Innovation,^114^ or Science of the Total Environment^115^ but do require research data to be provided or inclusion of a statement about its availability. Emerging Contaminants^116^ and Environmental Chemistry & Ecotoxicology^117^ are published jointly by Elsevier and China Science Publishing through KeAi and recommend similar reporting practices. However, Environmental Chemistry & Ecotoxicology also requires authors to provide a “stereochemistry abstract,” which includes all available stereochemical information for every chiral compound.^117^ No additional details or requirements for nonchiral substances are mentioned.

The Royal Society of Chemistry (RSC) has taken a distinctive approach that follows the recommendations of Schymanski and Bolton.^5^ For articles published in any of their journals, including Environmental Science: Atmospheres, Environmental Science: Nano, Environmental Science: Processes & Impacts, and Environmental Science: Water Research & Technology, they recommend including a summary file that contains chemical structural information such as the SMILES, InChI or InChIKey, chemical names and synonyms and any relevant metadata.^118^

These four publishers currently host the most relevant journals in environmental sciences. While all publishers encourage data transparency principles and provide options for authors to submit Supporting Information, most provide little to no guidance on how to share their data. To facilitate data transparency, publishers should provide more guidelines to authors on how to report chemical data to improve data FAIR-ness. The next section discusses how to share chemical information critical to advancing environmental sciences.

## 5 Recommendations for Data Reporting

Schymanski and Bolton^119^ note that *CSV* (comma-separated value) files are the most interoperable file format available for sharing Supporting information, including chemical information in an interdisciplinary context (i.e., where not all professionals are data scientists or informaticians). These files are easily readable by coding languages and different file systems and through the use of commonly available software such as Microsoft Excel or Google Sheets, and simple free text editors like the NotePad (Windows) or TextEditor (Apple iOS). Similarly, data can be shared in a *TSV* (tab-separated value) file.^119^ Both *CSV* and *TSV* file formats are easily read by a large number of free- and subscription-based software tools. The chemistry-specific formats, such as *SDF* or *MOL*, require external tools and packages to convert the data into a more user-friendly format and can be daunting for those less familiar with chemistry formats. When working with a large number of chemicals, *SDFs* can become very large and cumbersome to open and manipulate. However, *SDF* and *MOL* files allow for the transfer of 3D structural information of the chemical, which is difficult to do with other chemical identifiers reported in *CSV* or *TSV* files. For this reason, the use of *CSV* or *TSV* files is encouraged when reporting any chemical data, supplemented with *SDF* or *MOL* files as needed or if already available. Table SI 1 contains the chemical identifiers for all substances mentioned in this text and SI (see Section SI 1).

The file should contain clearly defined headers (*CSV*/*TSV*) or tags (*MOL*/*SDF*) and at least one chemical identifier: SMILES, InChI, a name, or InChIKey according to Schymanski and Bolton.^5^ It is best practice to avoid using spaces in the header names. Spaces can be replaced with “_” to maintain readability and reduce errors when reading files into external programs. Standardizing and using the FAIR Chemical Structures Template,^5^ with consistent column or header names helps cheminformaticians and data scientists to consolidate information to sync with databases and registries, without the need for additional data mapping.

To report novel chemicals accurately, at least two chemical identifiers should be reported, including at least one of SMILES or InChI. Any number of additional chemical identifiers that indicate common or trademarked names and database numbers (CAS RNs, CIDs, etc.) can also be included at the discretion of the author. It is also important to define the primary identifier (i.e., the most reliable of the information given) to help resolve occurrences of mismatching information (see Table SI 1). Having multiple sources of information about the chemical identity reduces the possibility of misidentification; defining the primary piece of information helps resolve potential clashes that remain. Since, for instance, CAS RNs can only be reliably obtained from a closed database (i.e., SciFinder^59^) and publicly available mappings may be absent or prone to errors, it is useful to know whether this is the primary identifier during curation. A thorough description of the substance studied has the potential to improve the usability of the research when working with more complex scenarios, such as mixtures and polymers. Authors of all environmentally related disciplines are encouraged to adopt these best practices for reporting chemical information, and environmental science journals are encouraged to adopt minimum reporting standards for chemical identification (Box [Boxed-text box2]). These recommendations align with those made in an IUPAC Technical Report for good reporting practices of physical–chemical property measurements.^120^

2 Recommendations for Reporting Chemical Information

**File Format:**

● *CSV* or *TSV* file
● *MOL* or *SDF* if the 3D conformation of the substance is important

**Chemical Identifiers (minimum 2), indicating primary identifier:**

● Structural identifier (minimum 1):
  – SMILES
  – InChI
● Other Identifier (minimum 1):
  – Chemical name
  – Database identifier
  – Optionally:
    * InChIKey
    * Additional names (IUPAC, common, technical) or synonyms
    * Additional database identifiers (CAS RN, CID, DTXSID)
    * Drawing of structures

Obtaining the necessary identifiers depends on the certainty of the primary identifier. If the structure of the compound is available, the SMILES can be generated using drawing programs, which can then be used to search for the CAS RN on SciFinder^59^ or CAS Common Chemistry,^58^ or to search directly in CompTox^121^ or PubChem.^122^ Once a single database entry is found, verify that the record matches the specificity of the substance being characterized before extracting other chemical identifiers. If there is already a primary identifier associated with the substance, e.g., CAS RN, check first that the entry matches the chemical you expect on the database platform, in this case, CAS Common Chemistry^58^ or SciFinder.^59^ If there is no database or registry data available about the chemical, or the available information is not as ambiguous or as specific as required, the structural identifier can be generated directly, using tools such as Open Babel^11^ or using ACD/Labs ChemSketch.^10^ Erroneous or mismatched data found within a registry can be reported by email^122^ or through built-in error functions (e.g., ChemSpider^123^ and CompTox “Submit Comment”^121^). When working with multiple chemicals at once, many tools, including CompTox,^121^ PubChem,^122^ and Open Babel,^11^ are capable of running batch searches or conversions using chemical identifiers. Most registries also provide an Application Programming Interface (API) access to their data.

This paper focuses on guidelines for reporting chemical information; however, to improve FAIR practices and increase the applicability and rigor of scientific research, authors should aim to provide additional information regarding experimental and modeling data that they generate and use. Previous publications have provided guidance on reporting data related to environmental exposure,^124^ plant bioaccumulation test results,^125^ mass spectrometry^126^ and property measurements.^120^ If something was considered during the research design phase, it is likely something that others in the field or related fields would be interested in.

Ideally, all research articles related to chemistry in the environmental sciences should have at least one Supporting Information text or document containing a summary of the chemicals discussed. Almost all journal publications are entirely digital and provide options to include one or more SI documents. Additionally, the SI can be included as part of a preprint copy on ChemRxiv or on another free repository such as re3data^127^ (recommended by SETAC Journals^110^), Zenodo,^128^ or FigShare.^129^ In some instances, it is not possible to submit *CSV* or *TSV* files as supplementary documentation. As an example, Table SI 1 is available on Zenodo at 10.5281/zenodo.14931110 as a *CSV* file and as part of the Supporting Information as an *XLSX* file. Sharing data and providing detailed SI are extremely useful to other researchers and allow the data to have better reach and a larger impact on future research.

The inclusion of Supporting Information with chemical data makes the data within the research article more findable and accessible to the reader. It prevents and reduces errors associated with the misidentification of chemicals, allowing data presented in the articles to be more readily used in future research, whether that involves building new models, designing experiments, or developing methods. Presenting data in consistent and clear formats helps make the integration of data into larger data sets easier for researchers and data managers, exemplified in initiatives such as PubChem,^52,53^ CompTox,^54^ and the NORMAN Suspect List Exchange^130^ (NORMAN-SLE). A further example of this includes data-driven approaches such as machine learning (ML) and artificial intelligence (AI), which require readily (re)­usable data sets. If data is not made readily reusable, it is logical to assert that it will slow the rate of discovery and innovation within the fields of environmental science, chemistry, and toxicology.

The ultimate goal of scientific publishing is to disseminate findings and research. By doing so, we influence and impact future research and facilitate further developments in science and society. Therefore, the usefulness of results relating to chemical data is often dependent on the meta information that is made available and how well the chemical is identified. Results from exposure experiments investigating the toxicity or bioaccumulation of a chemical must be interpreted within the context of the identity, purity, concentration, and properties of the chemicals used. Environmental observations and measurements of chemicals can be used by other researchers to verify their own work and prioritize and deprioritize chemicals of emerging concern. Physical–chemical and toxicity data are useful in exposure and risk assessment, monitoring, experimental design, and the development and verification of quantitative structure–property-activity relationships (QSPR/QSAR).

During the days of print-only journals, the length of a journal article was far more restricted, and thus detailed reporting on the data and methodologies was often very limited. However, with the move to digital publishing, open access platforms, and data sharing, these restrictions no longer exist. Instead, we are confined only by journal and copyright limitations and our capacity and willingness to share data.

## Supplementary Material





## Data Availability

Table SI 1 contains chemical information for all chemicals mentioned or referenced in this text and the SI as a *CSV* file on Zenodo at 10.5281/zenodo.14931110.

## Notes

Additional details, figures, and tables (citing refs 10,18,24 , and^131^) (PDF)
Chemical information for all chemicals mentioned or referenced in this text (Table SI 1) (XLSX)
