# Supplementary material for: Reporting Chemical Data in the Environmental Sciences
Source: ACS Environ Au. 2025 Jul 29;5(5):444–56. doi: 10.1021/acsenvironau.5c00034 (PMC12447227; doi:10.1021/acsenvironau.5c00034)
Supplement: Supplementary file 1 [file vg5c00034_si_001.pdf]

Supporting Information: Reporting Chemical Data in the Environmental Sciences

Sivani Baskaran,[*,†] Parviel Chirsir,[‡] Shira Joudan,[¶] Raoul Wolf,[†] Evan E. Bolton,[§] Paul A. Thiessen,[§] and Emma L. Schymanski[‡]

†*Norwegian Geotechnical Institute (NGI), 0484, Oslo, Norway*

‡*Luxembourg Centre for Systems Biomedicine (LCSB), University of Luxembourg, 6 Avenue du Swing, 4367 Belvaux, Luxembourg*

¶*Department of Chemistry, University of Alberta, Edmonton, Alberta, T6G 2G2 Canada*

§*National Center for Biotechnology Information (NCBI), National Library of Medicine (NLM), National Institutes of Health (NIH), Bethesda, Maryland 20894, United States*

E-mail: sivanibaskaran1@gmail.com

Contents

SI 1 Chemical information for substances reported **S-2**

SI 2 Importance of stereochemistry & specificity **S-2**

SI 3 Explaining SMILES notation **S-6**

References **S-9**

SI 1 Chemical information for substances reported

Chemical information for all substances mentioned in this article is made available in the attached *XSLX* file (Table SI 1) available as part of the supporting documentation and on Zenodo as a *CSV* file (10.5281/zenodo.14931110). The file includes the SMILES, InChI, InChIKey and some of the different names used to describe the substances. Where possible the PubChem CIDs, CompTox DTXSIDs, and CAS RNs are also provided. All drawings for chemical structures provided in the main text and the SI were made using ACD/ChemSketch.[S1]

As much of the details for hexabromocyclododecane (HBCD) isomers are not well documented on open platforms, a structure similarity search in SciFinder was used to identify all possible isomers. A similar search was applied to PubChem and CompTox (using the Beta version of substructure search) to identify their corresponding entries. The name (with R/S specificity), InChI and InChIKey were used to identify the record associated with each substance in CompTox and PubChem. If R/S specificity was not included in the names, the InChI was used to match the substance to our list. The InChI and InChIKey did not always match each other in the CompTox Dashboard.

Structures for the enantiomers of 6PPD-quinone were not found in the cited literature or databases and registries. To identify the structure of (*R*)-6PPD-quinone and (*S*)-6PPD-quinone, the structure of the 6PPD-quinone was drawn in ACD/ChemSketch[S1] using the Stereoisomer Generator and Generate Stereo Descriptors features to identify the *R* and *S* enantiomers.

SI 2 Importance of stereochemistry & specificity

As discussed in Section 2.3, the same standard InChI string can describe multiple tautomers of the same structure. In some instances, researchers may consider these different tautomers to be different chemicals. With SMILES, the exact structure can be described in a single-line notation. When working with standardized InChI strings it is possible to lose information

regarding the specific position of hydrogens.[S2] Non-standard InChI with fixed hydrogens will be distinct between tautomers.[S2] This is discussed in detail by Heller et al..[S2] Table SI 2 shows five possible tautomers for atrazine and how they can be distinguished using SMILES notation; the first is the most environmentally stable and relevant structure.

Table SI 2: Five different tautomers for atrazine described using chemical structure drawing and the SMILES. All structures have the same standard InChI: InChI=1S/C8H14ClN5/c1-4-10-7-12-6(9)13-8(14-7)11-5(2)3/h5H,4H2,1-3H3,(H2,10,11,12,13,14) and InChIKey: MXWJVTOOROXGIU-UHFFFAOYSA-N. The first structure in the table is the most environmentally relevant/stable.

Structure	SMILES
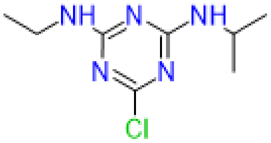	C(NC1=NC(=NC(=N1)Cl)NC(C)C)C
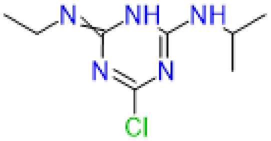	C(N=C1NC(=NC(=N1)Cl)NC(C)C)C
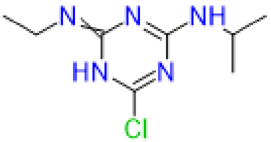	C(N=C1N=C(N=C(N1)Cl)NC(C)C)C
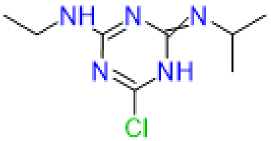	C(NC1=NC(NC(=N1)Cl)=NC(C)C)C
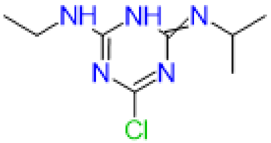	C(NC1=NC(=NC(N1)=NC(C)C)Cl)C

Section 2.1 and 2.2 briefly explored the example of hexabromocyclododecane (HBCD), a 12-carbon ring with six bromine substitutions. The structural isomer, 1,2,5,6,9,10-HBCD and its 16 possible stereoisomers are shown in Figure SI 1 and Figure SI 2. Pairs of enantiomers are presented together. In registries and databases, the two enantiomers are sometimes defined based on optical activity, noted with + or - (see Table SI 1).[S3] For example, the two alpha-HBCDD enantiomers are collectively referred to as (±)-alpha-HBCD or individually as (+)-alpha-HBCD and (-)-alpha-HBCD. Where the mirror image does not produce a unique

stereochemistry, no notation is required, *e.g.*, delta-HBCD. The determination of optical activity must be made experimentally,[S4] thus this naming convention is not available for all enantiomer pairs (see Table SI 1).

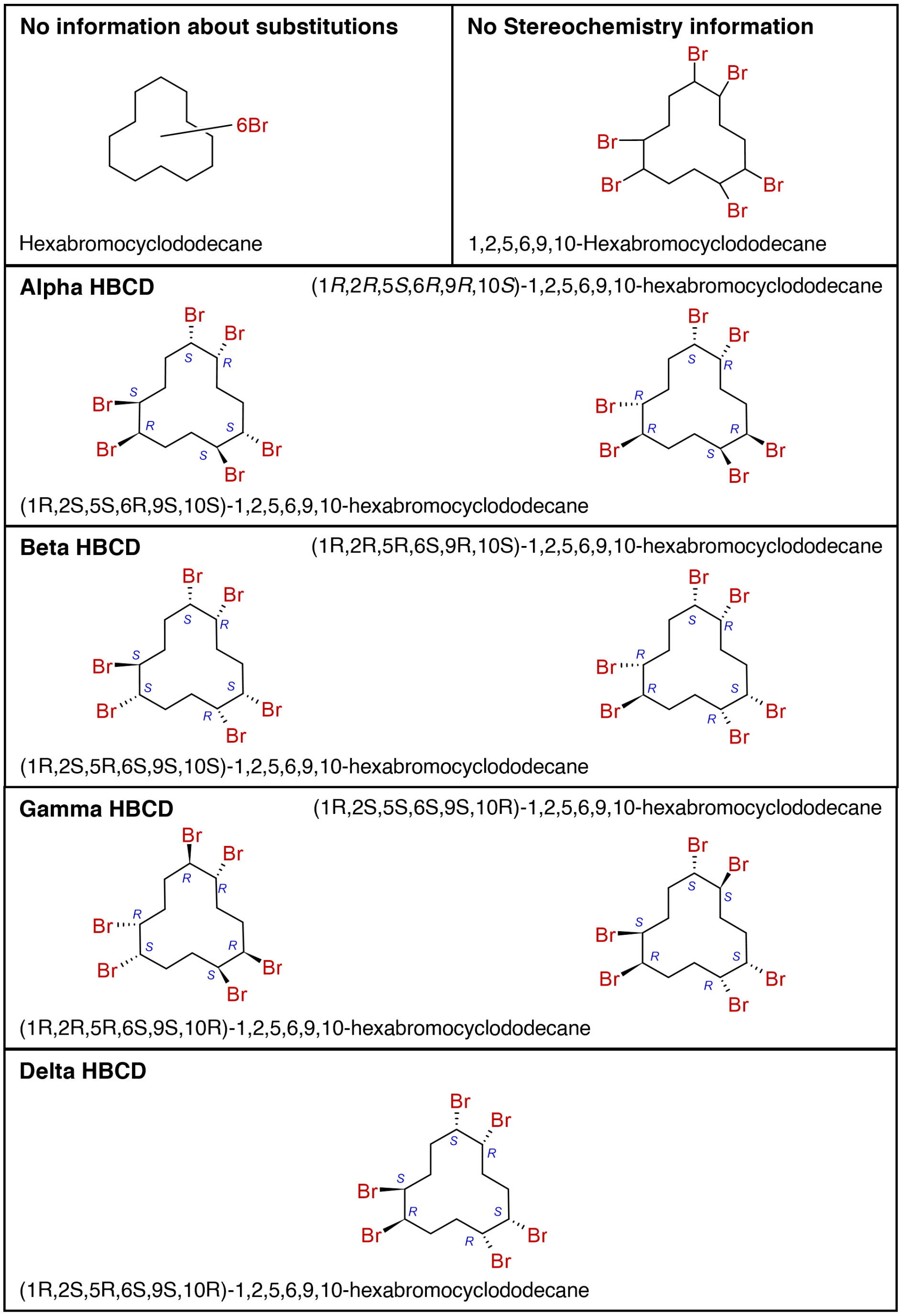

Figure SI 1: Generic HBCD, non-stereo specific 1,2,5,6,9,10-HBCD, and some stereoisomers of 1,2,5,6,9,10-HBCD. Continued in Figure SI 2.

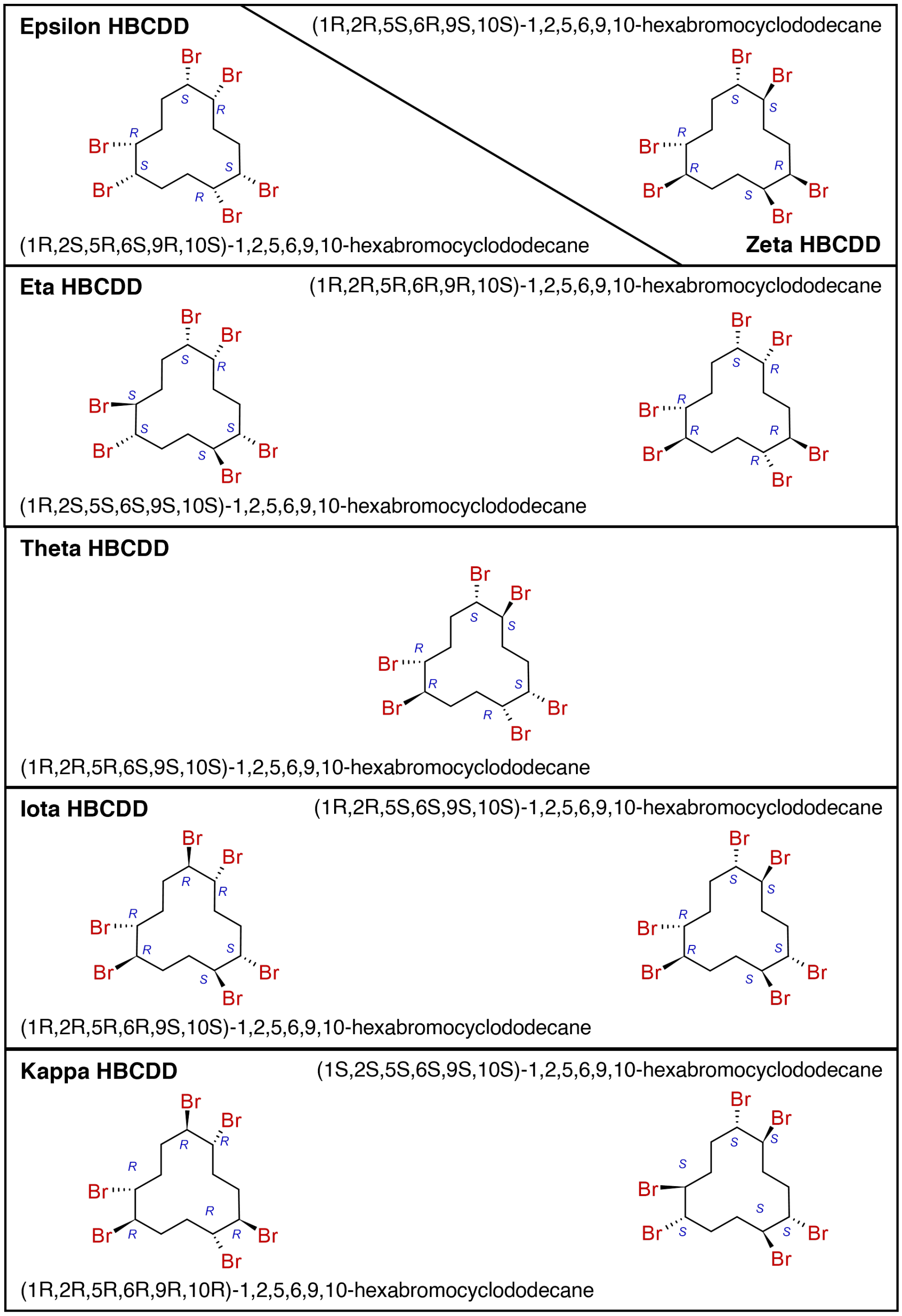

Figure SI 2: Continued from Figure SI 1, stereoisomers of 1,2,5,6,9,10-HBCD.

SI 3 Explaining SMILES notation

Figure 1 shows the difference in SMILES notation for *E* and *Z* stereochemistry and chirality. Compound D and Substance B are used as examples in Figure SI 3 to show how the same chemical can be depicted in different ways.

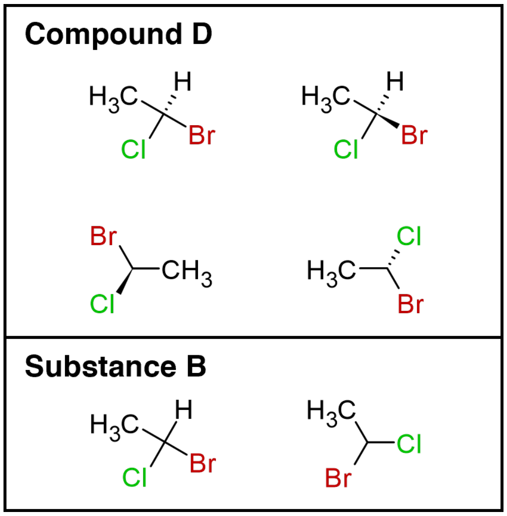

Figure SI 3: Different structural representations of Compound D and Substance B from Figure 1.

Figure SI 4 includes additional examples which depict the difference between single, double, and triple bonds in SMILES (Part A), dative and non-dative bond structures and SMILES (Part B), and kekulized and non-kekulized aromatic rings (Part C). While it is difficult to visualize a larger structure by looking at the SMILES string, identifying key features can help reduce larger errors and be used as a quick check for the validity of the SMILES string.

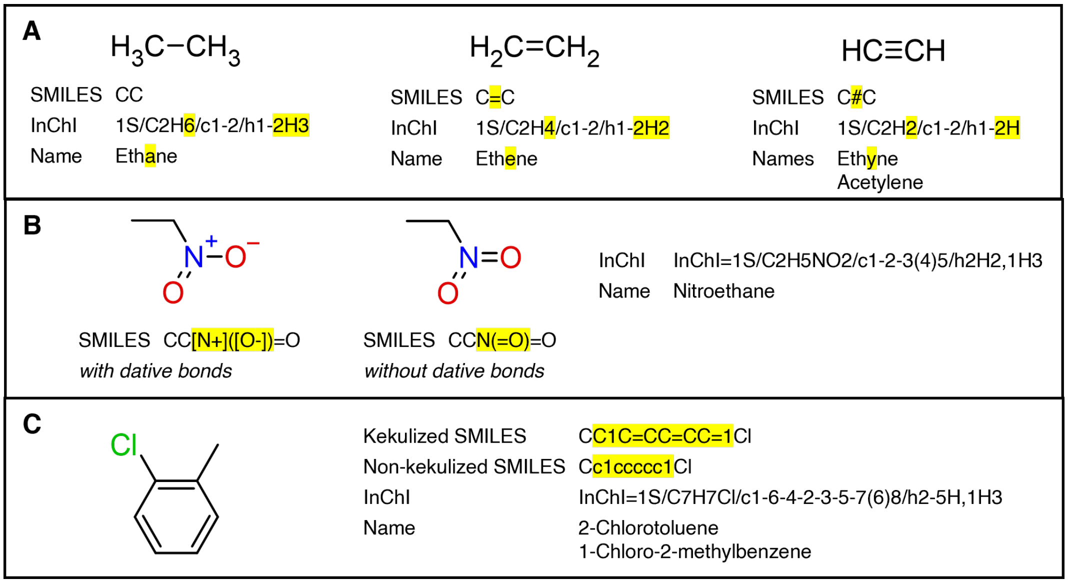

Figure SI 4: A. Examples of single, double, and triple bonds represented in SMILES notation; B. Example of a dative and non-dative chemical structure and SMILES notation for the same compound; C. Kekulized and non-kekulized SMILES notation for the same structure.

It is possible to identify SMILES notation for a mixture or salt complex. When there are multiple components, each is separated by a "." in the SMILES and the connectivity layer of an InChI string. This is visible in Figure SI 5, where chemical identifiers for select PFOS salts are depicted.

Perfluorooctanesulfonate
CAS RN: 45298-90-6
DTXSID: DTXSID80108992
SMILES: [O-]S(=O)(=O)C(F)(F)C(F)(F)C(F)(F)C(F)(F)C(F)(F)C(F)(F)C(F)(F)C(F)(F)F
InChI=1S/C8HF17O3S/c9-1(10,3(13,14)5(17,18)7(21,22)23)2(11,12)4(15,16)6(19,20)8(24,25)29(26,27)28/h(H,26,27,28)/p-1

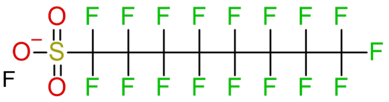

Lithium perfluorooctanesulfonate
CAS RN: 29457-72-5
DTXSID: DTXSID2032421
CID: 23677927
SMILES: [Li+].[O-]S(=O)(=O)C(F)(F)C(F)(F)C(F)(F)C(F)(F)C(F)(F)C(F)(F)C(F)(F)C(F)(F)F
InChI=1S/C8HF17O3S.Li/c9-1(10,3(13,14)5(17,18)7(21,22)23)2(11,12)4(15,16)6(19,20)8(24,25)29(26,27)28;/h(H,26,27,28);/q;+1/p-1

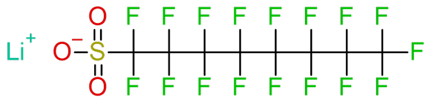

Potassium perfluorooctanesulfonate
CAS RN: 2795-39-3
DTXSID: DTXSID8037706
CID: 23669238
SMILES: [K+].[O-]S(=O)(=O)C(F)(F)C(F)(F)C(F)(F)C(F)(F)C(F)(F)C(F)(F)C(F)(F)C(F)(F)F
InChI=1S/C8HF17O3S.K/c9-1(10,3(13,14)5(17,18)7(21,22)23)2(11,12)4(15,16)6(19,20)8(24,25)29(26,27)28;/h(H,26,27,28);/q;+1/p-1

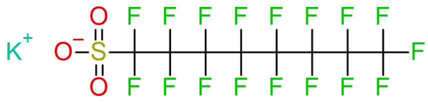

Ammonium perfluorooctanesulfonate
CAS RN: 29081-56-9
DTXSID: DTXSID9067435
CID: 15607692
SMILES: [NH4+].[O-]S(=O)(=O)C(F)(F)C(F)(F)C(F)(F)C(F)(F)C(F)(F)C(F)(F)C(F)(F)C(F)(F)F
InChI=1S/C8HF17O3S.H3N/c9-1(10,3(13,14)5(17,18)7(21,22)23)2(11,12)4(15,16)6(19,20)8(24,25)29(26,27)28;/h(H,26,27,28);1H3

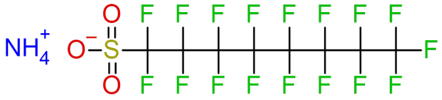

Triethylammonium perfluorooctanesulfonate
CAS RN: 54439-46-2
DTXSID: DTXSID101033033
CID: 138489998
SMILES: CC[NH+](CC)CC.[O-]S(=O)(=O)C(F)(F)C(F)(F)C(F)(F)C(F)(F)C(F)(F)C(F)(F)C(F)(F)C(F)(F)F
InChI=1S/C8HF17O3S.C6H15N/c9-1(10,3(13,14)5(17,18)7(21,22)23)2(11,12)4(15,16)6(19,20)8(24,25)29(26,27)28;1-4-7(5-2)6-3/h(H,26,27,28);4-6H2,1-3H3

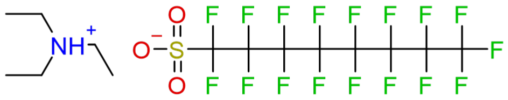

Figure SI 5: Perfluorooctanesulfonate and some of its salts.

References

(S1) ChemSketch. ACD/Labs, 2023; https://www.acdlabs.com/resources/free-chemistry-software-apps/chemsketch-freeware/, Accessed: 2025-01-15.

(S2) Heller, S. R.; McNaught, A.; Pletnev, I.; Stein, S.; Tchekhovskoi, D. InChI, the IUPAC International Chemical Identifier. *J. Cheminf.* **2015**, *7*, 23, DOI: 10.1186/s13321-015-0068-4.

(S3) International Union of Pure and Applied Chemistry (IUPAC) Optical Activity. *IUPAC Compendium of Chemical Terminology* **2006**, DOI: 10.1351/goldbook.C01039.

(S4) Huang, R.; Lin, Z.; Liu, Y.; Wu, X.; Yuan, K. A "Hand-Held" Polarimeter for on-Site Chiral Drug Measurement and Chemical Reaction Monitoring. *Anal Bioanal Chem* **2025**, *417*, 1055–1065, DOI: 10.1007/s00216-024-05729-4.
